# A *KCNB1* gain of function variant causes developmental delay and speech apraxia but not seizures

**DOI:** 10.3389/fphar.2022.1093313

**Published:** 2022-12-21

**Authors:** Emma L. Veale, Alessia Golluscio, Katheryn Grand, John M. Graham, Alistair Mathie

**Affiliations:** ^1^ Medway School of Pharmacy, University of Kent and University of Greenwich, Chatham Maritime, United Kingdom; ^2^ Department of Pediatrics, Harbor-UCLA Medical Center, Cedars-Sinai Medical Center, David Geffen School of Medicine at UCLA, Los Angeles, CA, United States; ^3^ School of Engineering, Arts, Science and Technology, University of Suffolk, Ipswich, United Kingdom

**Keywords:** Kv2.1 channel, KCNB1, pathogenic variant, developmental delay, gain of function, guanxitoxin-1E

## Abstract

**Objective:** Numerous pathogenic variants in *KCNB1*, which encodes the voltage-gated potassium channel, K_V_2.1, are linked to developmental and epileptic encephalopathies and associated with loss-of-function, -regulation, and -expression of the channel. Here we describe a novel *de novo* variant (P17T) occurring in the K_V_2.1 channel that is associated with a gain-of-function (GoF), with altered steady-state inactivation and reduced sensitivity to the selective toxin, guanxitoxin-1E and is clinically associated with neurodevelopmental disorders, without seizures.

**Methods:** The autosomal dominant variant was identified using whole exome sequencing (WES). The functional effects of the *KCNB1* variant on the encoded K_V_2.1 channel were investigated using whole-cell patch-clamp recordings.

**Results:** We identified a *de novo* missense variant in the coding region of the *KCNB1* gene, c.49C>A which encodes a p.P17T mutation in the N-terminus of the voltage-gated, K_V_2.1 potassium channel. Electrophysiological studies measuring the impact of the variant on the functional properties of the channel, identified a gain of current, rightward shifts in the steady-state inactivation curve and reduced sensitivity to the blocker, guanxitoxin-1E.

**Interpretation:** The clinical evaluation of this *KCNB1* mutation describes a novel variant that is associated with global developmental delays, mild hypotonia and joint laxity, but without seizures. Most of the phenotypic features described are reported for other variants of the *KCNB1* gene. However, the absence of early-onset epileptic disorders is a much less common occurrence. This lack of seizure activity may be because other variants reported have resulted in loss-of-function of the encoded K_V_2.1 potassium channel, whereas this variant causes a gain-of-function.

## Introduction

K_V_2.1 (Shab-related subfamily, member 1) is a voltage-gated potassium channel protein which, in humans, is encoded by the *KCNB1* gene (Alexander et al., 2021). K_V_2.1 channels give rise to a predominant delayed rectifier current (*I*_K_) that regulates action potential duration, firing frequency and neuronal excitability (Misonou et al., 2005). K_V_2.1 channels are widely expressed in the brain and are the main determinants of *I*_K_ currents in several neurons including cortical and hippocampal pyramidal cells and cerebellar granule neurons (Johnson et al., 2019) where their activity contributes to pro-apoptotic events (Shah & Aizenman 2014). K_V_2.1 channels are also found in the heart, pancreas, pulmonary arteries, auditory outer hair cells and the retina (Johnson et al., 2019).

**FIGURE 1:**
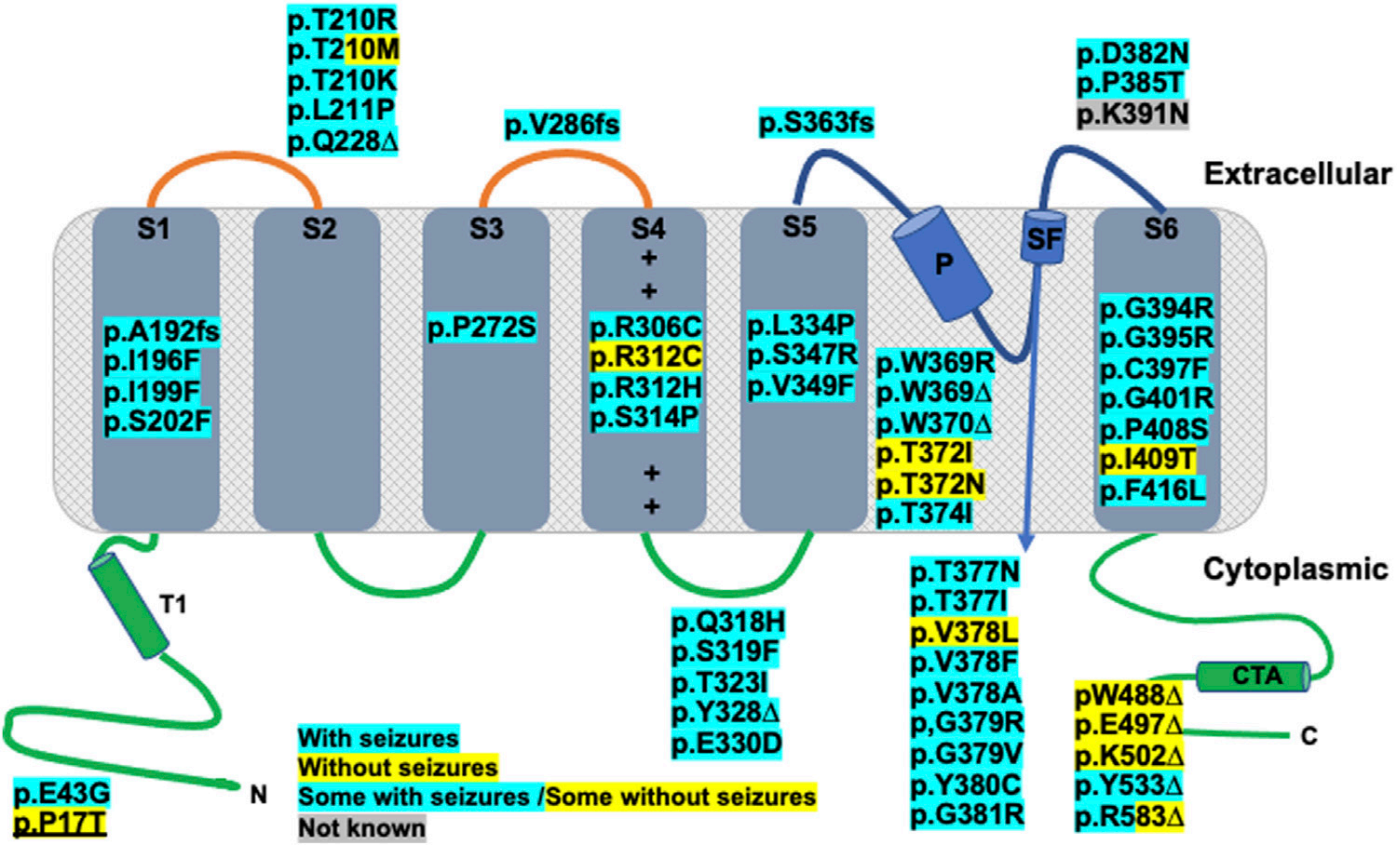
Kv2.1 protein α-subunit topology schematic representation, adapted from Bar et al., 2020 showing location of published and novel (underlined) clinical variants. Functional topology of single α-subunit consists of an N-terminal domain (N), residues 1–186, containing a tetramerization domain (T1), six transmembrane domains: S1 (residues 187–208), S2 (residues 229–250), S3 (residues 260–280), S4 (residues 295–316. + corresponds to positively charged amino acids of voltage sensor), S5 (residues 331–351), S6 (residues 392–420), pore helix (P), residues 365–376 and selectivity filter (SF), residues 377–381 and C-terminal domain (C) and tetramerization domain (CTA), residues 421–858. Truncating variants are indicated with an *∆*, whilst frameshift mutations are indicated with fs. All other mutations are missense. Any variants highlighted with blue correspond to patients with seizures, those highlighted yellow correspond to patients without seizures and blue/yellow corresponds to patients with and patients without seizure. For the variant highlighted grey, seizure data was not available.

Each K_V_2.1 channel is composed of four alpha-subunits, which can either be homomeric or form heteromeric complexes with other K_V_ channels including “silent” subunits (Salinas et al., 1997) such as, for example, with K_V_9.3 in cerebral arterial myocytes (O’Dwyer et al., 2020). Each alpha-subunit of a K_V_2.1 channel is comprised of six transmembrane spanning regions (S1 to S6), with a pore (P) loop between S5 and S6, (the S5-S6 linker) which incorporates the selectivity filter of the channel, while the voltage sensor is found on S4 (see Yellen 2002; Figure 1). The long N- and C- termini are located on the cytoplasmic side of the membrane and play a crucial role in the activation and inactivation of the channel (Ju et al., 2003).

Mutations of K_V_2.1 channels underlie epileptic encephalopathies, infantile epilepsy, autism and other neurodevelopmental disorders in identified individuals (Torkamani et al., 2014; Saitsu et al., 2015; Thiffault et al., 2015; Calhoun et al., 2017). More recent studies have extended identification of K_V_2.1 variants to 74 patients with 55 distinct missense or loss of function mutations (de Kovel et al., 2017; Bar et al., 2020; Xiong et al., 2022). 85% of the patients examined (62/73) developed epilepsy and all examined (73/73) had developmental delays, albeit with varying degrees of severity (de Kovel et al., 2017; Bar et al., 2020; Xiong et al., 2022).

In this study, a 5 years old boy with neurodevelopmental delay, speech apraxia, normal growth, normal EKG, and normal EEG with no seizures, was found to have a *de novo* variant in *KCNB1* (p.P17T; c49C>A). Proline 17 is located in the N-terminal region of the K_V_2.1 channel, proximal to the inactivation domain, which is distinct from all but one of the mutations described above (de Kovel et al., 2017; Bar et al., 2020; Xiong et al., 2022, Figure 1). This is a non-conservative amino acid substitution likely to impact secondary protein structure and, as such, is predicted to be a likely pathogenic variant. Here, we describe the clinical phenotype of this patient and provide a detailed functional characterization of the P17T mutated K_V_2.1 channel.

## Materials and methods

### Exome sequencing and variant analysis

Exome sequencing was carried out by GeneDx. Using genomic DNA from the proband and parents, the exonic regions and flanking splice junctions of the genome were captured using the SureSelect Human All Exon V4 (50 Mb), the Clinical Research Exome kit (Agilent Technologies, Santa Clara, CA) or the IDT xGen Exome Research Panel v1.0 (Integrated DNA Technologies, Coralville, IA). Massively parallel (NextGen) sequencing was done on an Illumina system with 100 bp or greater paired-end reads. Reads were aligned to human genome build GRCh37/UCSC hg19 and analyzed for sequence variants using a custom-developed analysis tool. A prediction tool, PROVEAN, (https://www.jcvi.org/research/provean), was used to assist in interpretation. Additional sequencing technology and the variant interpretation protocol has been previously described (Retterer et al., 2016). The general assertion criteria for variant classification are publicly available on the GeneDx ClinVar submission page (http://www.ncbi.nlm.nih.gov/clinvar/submitters/26957/).

### Mammalian expression plasmids and mutagenesis

Human full-length wild-type (WT) voltage-gated channel, *Shab*-related subfamily, member 1, K_V_2.1 (*KCNB1*, Genbank™ NM_004975 cDNA incorporated into pCMV6-XL4 vector (Cambridge Bioscience, Cambridge, United Kingdom) and human voltage-gated channel modifier subfamily S member 3 (*KCNS3*, K_V_9.3), Genbank™ NM_002252 incorporated into pCMV6-XL5 (OriGene Techologies, Inc. United States) was utilised. The reporter plasmid, Green Fluorescent Protein (GFP) incorporated in the pcDNA3.1 vector, was a kind gift of Helen Meadows, GlaxoSmithKline, United Kingdom. The clinically identified K_V_2.1 mutation, proline (P) to threonine (T) at position 17 (p.P17T; c49C>A, K_V_2.1_P17T) was introduced using the QuikChange kit (Agilent, CA, United States) as previously described (Veale et al., 2014a). All constructs were fully sequenced by DNA Sequencing and Services, MRC/PPU, University of Dundee, Scotland.

### Cell culture and transfection

All individual expression plasmids were transiently co-expressed along with the green fluorescent reporter gene GFP at a concentration of 0.5 µg per well (0.25 µg per well for the toxin experiments to allow for adequate voltage control), using a modified calcium-phosphate protocol, as previously described (Cunningham et al., 2019; Mathie et al., 2021) in a modified human embryonic kidney 293 cell line, tsA201 (European Collection of Authenticated Cell Cultures, Sigma-Aldrich, United Kingdom) prepared and maintained as previously described (Veale et al., 2014a; Cunningham et al., 2019; Mathie et al., 2021). For experiments where K_V_2.1 or K_V_2.1_P17T was co-expressed with the K_V_9.3 subunit a ratio of 0.5:0.5 µg was transfected.

### Electrophysiology solutions and compounds

Electrophysiological experiments were conducted using an extracellular solution comprising (in mM): 145 NaCl, 2.5 KCl, 2 MgCl_2_, 1 CaCl_2_ and 10 4-(2- hydroxyethyl)-1-piperazineethanesulfonic acid (HEPES) and an intracellular solution comprising 150 KCl, 3 MgCl_2_, 5 EGTA and 10 HEPES, adjusted to pH 7.4 for both solutions. For extracellular solutions, containing 10 mM tetraethylammonium (TEA) an equimolar amount of NaCl was substituted. Guangxitoxin 1E (GxTx-1E) was purchased from Tocris (Bio-Techne Ltd., Abingdon, United Kingdom) and prepared in distilled water to a concentration of 10 µM and stored at −20°C until required. GxTX-1E was diluted directly into the extracellular solution prior to experimentation at a concentration of either 10 or 100 nM and cells were incubated in toxin for 20 min prior to recording.

### Electrophysiological recordings in tsA201 cells

**FIGURE 2:**
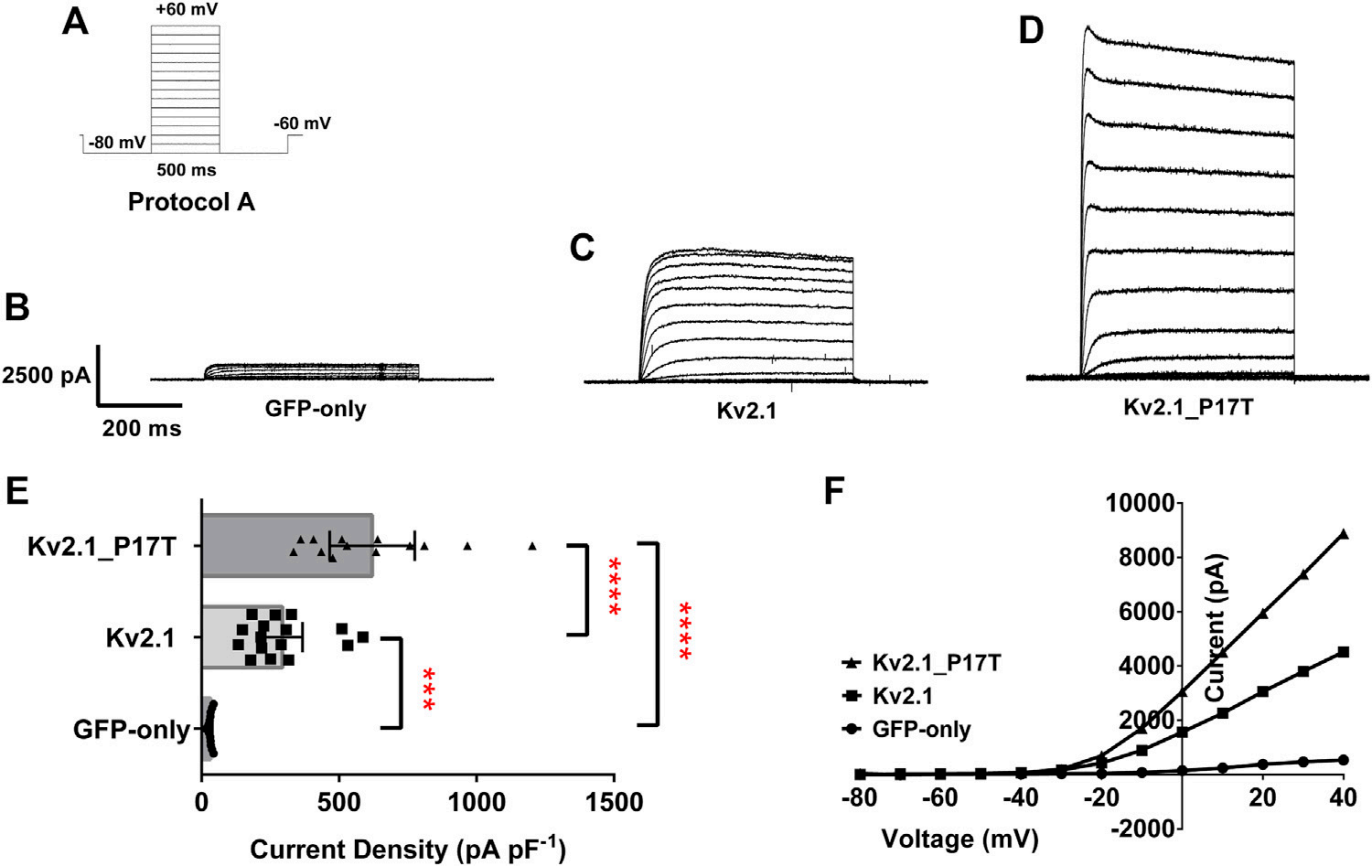
K_V_2.1 mutation, P17T is a gain of function mutation. **(A)** Schematic representation of voltage protocol A. tsA201 cells were held at −60 mV, stepped down to −80 mV for 500 m and then from −80 to +60 mV in 10 mV steps for 500 m, followed by a step back down to −80 mV for 500 m, then back to −60 mV. One sweep was recorded every 15 s, for 15 sweeps. **(B)** Average whole-cell current trace recorded using protocol A when expressing GFP-only.**(C)** Average whole-cell current trace recorded using protocol A when expressing K_V_2.1-WT. **(D)** Average whole-cell current trace recorded using protocol A when expressing K_V_2.1-P17T. **(E)** Histogram of current density (pA pF-1) measured at +50 mV from individual cells, transiently expressing 500 ng of GFP-only (*n* = 13), K_V_2.1-WT (*n* = 16) or K_V_2.1_P17T (*n* = 13). Error bars represent the 95% confidence intervals (CI). Statistical significance was tested using a one-way ANOVA with a post-hoc Dunnett’s multiple comparisons test, p < .01 for *** and p < 0.001 for ****. **(F)** Representative current voltage relationships for cells expressing GFP-only, K_V_2.1-WT or K_V_2.1-P17T.

**FIGURE 3:**
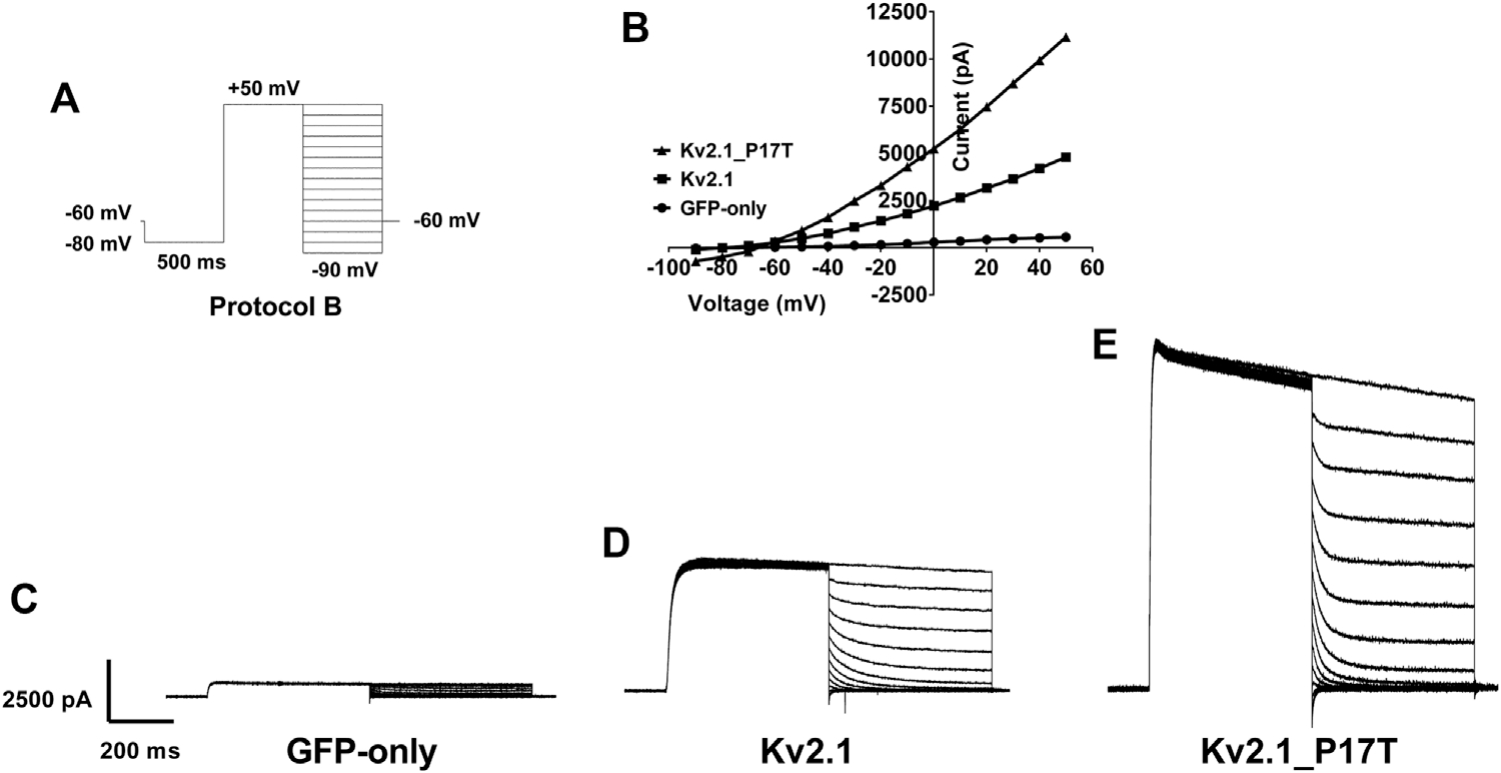
Reversal potential of currents through WT and K_V_2.1-P17T channels is unaltered. **(A)** Schematic representation of voltage protocol B. tsA201 cells were held at −60 mV, stepped down to −80 mV for 500 m then to +50 mV for 500 m. 10 mV steps from +50 to −90 mV for 500 milliseconds were then applied where the current was measured, then cells were stepped back to −60 mV, with one sweep every 15 s, for 15 sweeps.**(B)** Representative peak currents measured during the hyperpolarising current steps following current activation for cells expressing GFP-only, K_V_2.1-WT or K_V_2.1-P17T. Note that although the currents differ greatly in amplitude, they reverse direction at the same voltage. **(C)** Average whole-cell current trace recorded using protocol B when expressing GFP-only. **(D)** Average whole-cell current trace recorded using protocol B when expressing K_V_2.1-WT. **(E)** Average whole-cell current trace recorded using protocol B when expressing K_V_2.1-P17T.

Currents passing through WT or mutant channels transiently expressed in tsA201 cells were identified by their green fluorescence (excitation 395–440 nm, emission 470–600 nm) and were recorded using whole-cell patch clamp electrophysiology using three different protocols (A–C). Protocol A (see Figure 2A) consisted of cells being held at a holding potential of -60 mV followed by a step to −80 mV for 500-millisecond (ms) then cells were stepped from −80 mV to +60 mV in 10 mV steps, over 500 m, followed by a 500 m step to −80 mV before returning to the holding potential of −60 mV. Protocol B (see Figure 3A) consisted of cells being held at a holding potential of −60 mV followed by a step to −80 mV for 500 m then cells were stepped from +50 mV to −90 mV in 10 mV steps, over 500 m, followed by a 500 m step to −80 mV before returning to the holding potential of −60 mV. For both these protocols, individual sweeps were applied once every 15 s. For Protocol C, (see Figure 5A), cells were held at −80 mV, stepped to prepulse potentials from −100 mV to +10 mV in 10 mV increments for 30 s then to a test potential of +50 mV for 500 m. Cells were stepped back to 80 mV. For protocol C, sweeps were applied once every 70 s. Currents were recorded at room temperature (20–23^o^C) using an Axopatch 1D patch clamp amplifier (molecular Devices), low-pass filtered at 5 kHz before sampling (2–10 kHz).

### Electrophysiology data analysis and statistics

A measurement of current (picoampere, pA) for WT or the mutant channel was taken at the +50 mV step following the −80 mV prepulse of Protocol B and normalized to the cells membrane capacitance (picofarad, pF). Data acquisition was performed using pClamp 10.2 software (Axon Instruments). Data acquired was analysed using GraphPad Prism six software and Microsoft Excel and expressed as the mean ±95% Confidence Intervals (CI) and *n* represents the number of individual cells for each experimental condition. Where current amplitudes were used for comparisons, control currents (e.g., absence of toxin) were recorded on the same experimental days. Statistical comparisons were carried out using a one-way analysis of variance (ANOVA) with a post-hoc Dunnett’s multiple comparisons test. Data was considered statistically different *p* < .05 (*), *p* < .01 (**), *p* < 0.001 (***) and *p* < 0.0001 (****).

## Results

### Description of clinical phenotype

This patient was the full-term product of a normal pregnancy, labor and delivery born to a 30- year-old mother and a 39-year-old father. At 18.5 months old, he presented to our clinic due to global developmental delays. He had no intelligible speech, pulled to stand, took a few steps with support, army crawled, and scooted on his bottom. At 5 years 2 months, his height was 115 cm (85th percentile), with weight 18.5 kg (45th percentile), and head circumference 52.5 cm (81st percentile). His facial features were normal with no dysmorphic features. His speech articulation was compromised by a flaccid tongue that did not articulate speech sounds well. He had mild pectus excavatum, prominent fetal finger tip pads on his middle 3 fingers, and malalignment of his toes with incurving of his fourth and fifth toes, as well as incurving of his hallux and first toe. He had everted flat feet with a wide-based gait, mild joint laxity, good strength, and mild hypotonia. He had good social skills, a very short attention span, and a high level of activity. Developmental evaluation at age 34 months showed receptive language skills at 27–30 months, expressive language skills at 15–18 months, play skills at 30–33 months, interaction/attachment at 15–18 months, and social-emotional skills at 31 months. EKG and EEG were normal, and he has never had any seizures.

Exome sequencing revealed a *de novo* variant (p.Pro17Thr (CCG>ACG): c.49 C>A) in exon 1 in the *KCNB1* gene of this patient (Gene Dx #1859197). This individual’s mother (GeneDx #1859680) and father (GeneDx #1860837) do not harbour this P17T variant and sequence analysis and deletion testing of the mitochondrial genome in this patient was negative.

The P17T variant in the *KCNB1* gene has not been reported previously. This variant is a non-conservative amino acid substitution, which is likely to impact secondary protein structure as these residues differ in polarity, charge, size and/or other properties. In-silico analyses (see Methods), suggested that P17T is a likely pathogenic variant, consistent with the global developmental delay and hypotonia seen in this individual. These findings led us to investigate the functional properties of the P17T variant directly in this study.

### The P17T mutation enhances current through K_V_2.1 channels

Currents through homozygous human K_V_2.1-WT channels transiently transfected into human tsA-201 cells, were elicited by 10 mV steps in voltage from −80 to +60 mV (Protocol A, Figure 2A). This resulted in large voltage-dependent potassium currents, with characteristic outward rectification (Figures 2C, F). The average whole-cell current (pA) measured at +50 mV and normalised to cell capacitance (picofarads, pF) was 294 pA pF^−1^ (*n* = 16) 95% CI = 221 to 367 (Figure 2E). This was significantly different [*p* < 0.001, (95% CI = 111 to 412)] to cells expressing 500 ng GFP alone, (Figures 2B, E), which, when stimulated with the same voltage-protocol (Figures 2B, F), displayed small currents of 33 pA pF^−1^ (*n* = 13) 95% CI = 28 to 37 (Figure 2E). Untransfected (or GFP alone transfected) tsA201 cells naturally express a small background K_V_ component (see Figure 2B), which can be blocked by TEA (10 mM), significantly reducing (*p* < 0.0001, 95% CI: −34 to −17) the endogenous current to 7.4 pA pF^−1^ (*n* = 4) 95% CI = 0.4 to 14 (data not shown, see also Mathie et al., 2021).

Similar experiments with homozygous human K_V_2.1-P17T gave outward currents of 621 pA pF^−1^ at +50 mV, (*n* = 13) 95% CI = 466 to 775 (Figures 2D–F) that were significantly larger (*p* < 0.0001, 95% CI = −746 to −430, GFP-only and 95% CI = −477 to −176, K_V_2.1-WT) than both background and K_V_2.1-WT currents, respectively.

To investigate the effects of the mutant channel in a heterozygous background, we co-expressed K_V_2.1-P17T with K_V_2.1-WT in a 1:1 ratio. Co-expression of K_V_2.1-WT with K_V_2.1-P17T resulted in large outward currents of 707 pA pF^−1^ (*n* = 3) 95% CI = 76 to 1,338 that were not significantly different [*p* > .05, using a one-way ANOVA, Dunnett’s multiple comparisons test (95% CI = −494 to 377)] from the homozygous mutant channel (K_V_2.1-P17T) but were significantly different [*p* < 0.05, (95% CI = −750 to −8)] from homozygous K_V_2.1-WT (see also Supplementary Figure S1). Thus, in these co-expression experiments, K_V_2.1-P17T appears to be acting as a dominant-positive mutant channel subunit to increase the current through heteromers with K_V_2.1-WT channel subunits.

### The P17T mutation does not alter the ion selectivity of K_V_2.1 channels

A second voltage protocol was employed to determine the reversal potential of the K_V_2.1 WT and mutated channels. In this protocol (protocol B, Figure 3A), current was activated by a depolarising step to +50 mV before hyerpolarising steps in 10 mV increments were employed following the channel activation, in order to measure the currents’ reversal potential. From this protocol, it is clear that there is no difference in the reversal potential of currents evoked in untransfected cells (Figures 3B, C), cells transfected with K_V_2.1 (Figures 3B, D) or cells transfected with K_V_2.1-P17T (Figures 3B, E) showing that the mutated channel retains the K selectivity of the WT channel. This is an important observation, because certain mutations of K_V_2.1 (S347R, T374I, V378A, and G379R) do cause changes in reversal potential, indicative of effects on ion selectivity with a loss of selectivity for K^+^ over Na^+^ seen in these mutated channels (Torkamani et al., 2014; Thiffault et al., 2015). Furthermore, ion selectivity in other potassium channels in influenced by the N terminus. For example, in TREK-1 K2P channels (Veale et al., 2014b), alternative translation initiation produces a shorter N terminus truncated form of the channel with an altered ion selectivity.

### The P17T mutation also enhances current through K_V_2.1/K_V_9.3 heteromeric channels

We then investigated the effect of co-expressing the K_V_2.1 mutant channel with an electrically silent K_V_ channel, K_V_9.3, that has been shown, *in vitro*, to form heterotetramers with K_V_2.1 to modulate its electrophysiological and pharmacological properties (e.g., Patel et al., 1997; Kerschensteiner and Stocker, 1999). On their own, K_V_9.3 channels cannot get to the membrane and remain in the ER (Bocksteins 2016) and so do not evoke measurable functional currents (Patel et al., 1997).

**FIGURE 4:**
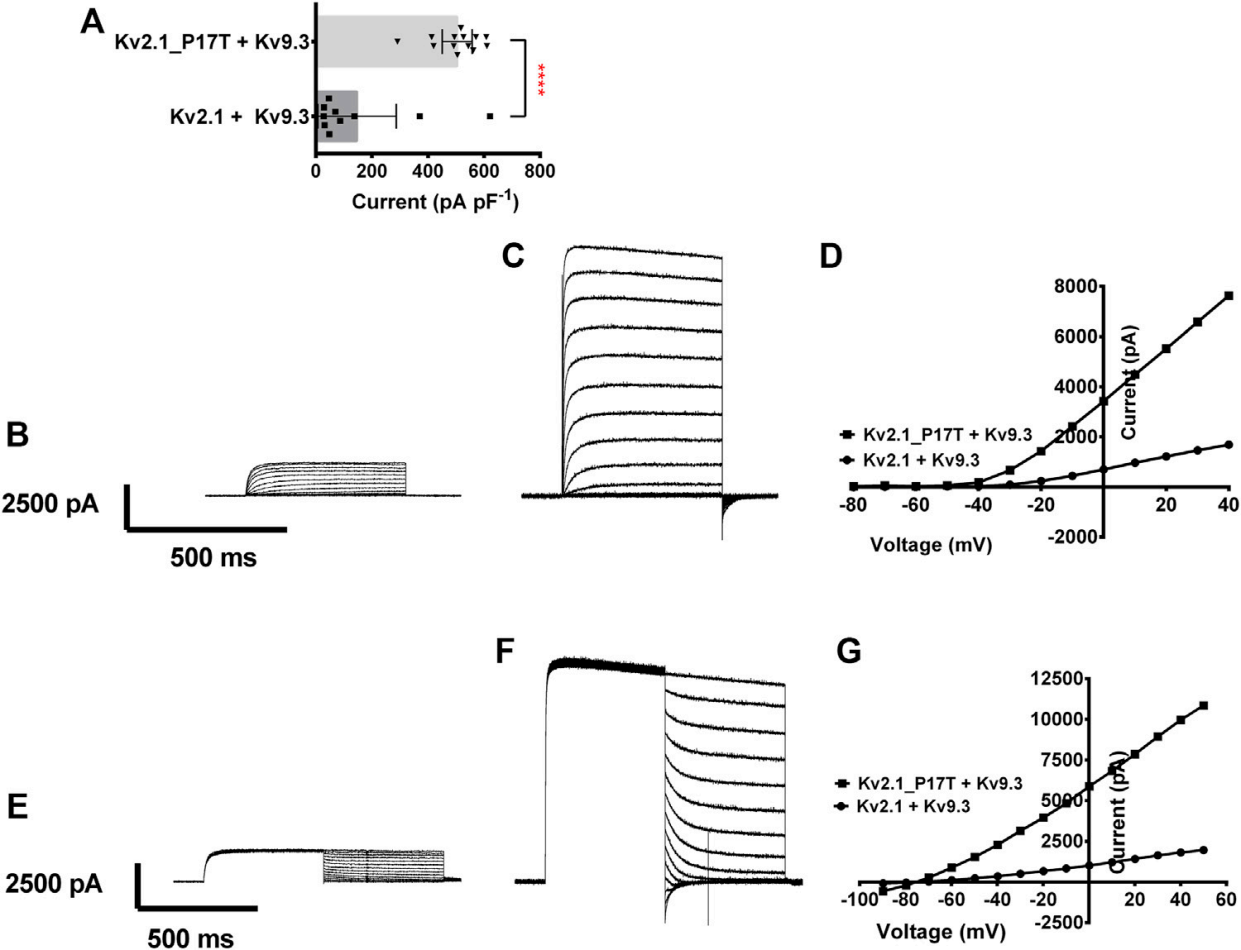
Coexpression of K_V_2.1-WT and K_V_2.1-P17T with K_V_9.3 shows gain of function for heteromeric channels. **(A)** Histogram of current density (pA pF^−1^) measured at +50 mV from individual cells, transiently expressing 500 ng of K_V_2.1-WT + K_V_9.3 (1:1, *n* = 8), or K_V_2.1-P17T + K_V_9.3 (1:1, *n* = 6). Error bars represent 95% CI. Statistical significance was tested using a Student’s *t* test, *p* < 0.001 for ****. **(B)** Average whole-cell current trace recorded using protocol A when expressing 500 ng K_V_2.1 + K_V_9.3 (1:1). **(C)** Average whole-cell current trace recorded using protocol A when expressing K_V_2.1-P17T + K_V_9.3 (1:1). **(D)** Representative current voltage relationships for cells expressing K_V_2.1-WT + K_V_9.3 (1:1), or K_V_2.1-P17T + K_V_9.3 (1:1). **(E)** Average whole-cell current trace recorded using protocol B when expressing 500 ng Kv2.1 + K_V_9.3 (1:1). **(F)** Average whole-cell current trace recorded using protocol B when expressing K_V_2.1-P17T + K_V_9.3 (1:1). **(G)** Representative peak currents measured during the hyperpolarising current steps following current activation with protocol B for cells expressing K_V_2.1-WT + K_V_9.3 (1:1), or K_V_2.1-P17T + K_V_9.3 (1:1).

Co-expression of K_V_9.3 (500 ng) with K_V_2.1-WT (500 ng) gave a current density of 95 pA pF^−1^ (*n* = 8) 95% CI = −3 to 193 (see Figures 4A, B, D). The current density of K_V_2.1-P17T when co-expressed with K_V_9.3 was 473 pA pF^−1^ (*n* = 6) 95% CI = 365 to 581 (see Figures 4A, C, D). Interestingly, K_V_2.1-P17T/K_V_9.3 current density is significantly larger than K_V_2.1-WT/K_V_9.3 [*p* < 0.0001 (95% CI = −665 to −90), see Figure 4A]. This suggests that K_V_2.1-P17T also acts as a dominant positive in the presence of Kv9.3 channels. Using protocol B, it is clear that there is no difference in the reversal potential of currents evoked in cells transfected with K_V_2.1-WT/K_V_9.3 (Figures 4E, G) or cells transfected with K_V_2.1-P17T/K_V_9.3 (Figures 4F, G) showing that the mutated channel retains the K selectivity of the WT channel in heteromultimers with K_V_9.3 subunits.

The P17T mutation alters the steady state inactivation of both K_V_2.1 and K_V_2.1/K_V_9.3 channels, such that more channels are available for activation at depolarised voltages.

Specific N-terminal interactions between K_V_2.1 and modulatory alpha-subunits such as K_V_9.3, promote the assembly of heterotetrameric channels (Post et al., 1996; Kramer et al., 1998; Stocker et al., 1999). However, these interactions can also alter the functional properties of the resulting channels when compared to WT K_V_2.1 channels. For example, the steady-state inactivation of K_V_2.1 channels is considerably altered in the presence of K_V_9.3 channel subunits (Kerschensteiner and Stocker, 1999; Kerschensteiner et al., 2003). Furthermore, a number of studies have suggested that amino acids in the N terminus of both K_V_2.1 (Lvov et al., 2008) and other K_V_ channels such as K_V_3.1 and K_V_1.5 (Klemic et al., 2001; Kurata et al., 2002; Kurata et al., 2005) are determinants of the slow “U-type” steady-state inactivation from closed states seen for these channels (Klemic et al., 1998), although other regions of these channels have also been shown to be important (Kurata & Fedida 2006; Coonen et al., 2020).

**FIGURE 5:**
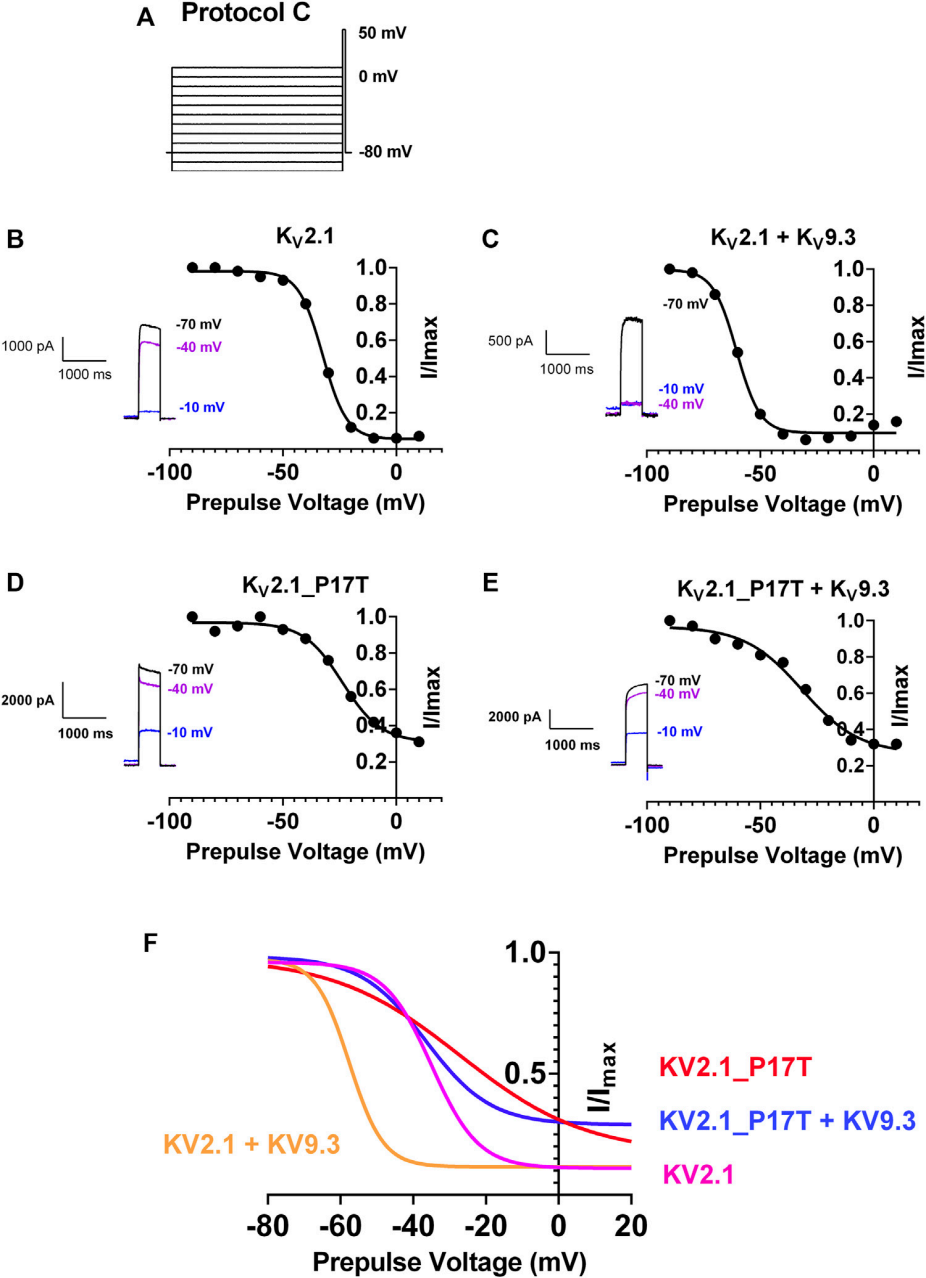
P17T mutation alters steady-state inactivation of both K_V_2.1 and K_V_2.1/K_V_9.3 channels. **(A)** Schematic representation of voltage protocol C. tsA201 cells were held at −80 mV, stepped to prepulse potentials from −100 mV to +10 mV in 10 mV increments for 30 s then to a test potential of +50 mV for 500 m. Cells were stepped back to 80 mV **(B)** graph of current recorded at +50 mV following a prepulse to the voltage indicated, normalised to the current seen when the prepulse was −90 mV, for K_V_2.1 channels. Solid line represents an unconstrained inverse Boltzmann fit to the data. Inset shows current steps at +50 mV following prepulses of −70, −40, and −10 mV **(C–E)** as for **(B)** for K_V_2.1/K_V_9.3 channels **(C)**, K_V_2.1_P17T channels **(D)** and K_V_2.1_P17T/K_V_9.3 channels **(E)** respectively. **(F)** Superimposition of Boltzmann curves from panels **(B–E)**.

To determine any effect of the P17T mutation on steady-state inactivation of both K_V_2.1 homomeric channels and K_V_2.1/K_V_9.3 heteromeric channels, we utilised a protocol (protocol C, Figure 5A) with a long conditioning prepulse (30 s) to various voltages before the test pulse (to +50 mV), in order to reach steady-state inactivation at each voltage before the same test pulse was applied (Kerschensteiner and Stocker, 1999).

For K_V_2.1 alone, unconstrained inverse Boltzmann fits to the data show that the V_50_ (50% steady state inactivation) occurs at −32.5 mV (95% CI = −33.5 to −31.5 mV, Figures 5B, F). This means that at a holding potential of −40 mV, say, much of the current is still available for activation (see Figure 5B inset). Co-expression with K_V_9.3 channels shifted the steady-state inactivation curve to the left, with a corresponding shift in V_50_ to −60.4 mV (95% CI = −62.7 to −58.3 mV, Figures 5C, F), so that virtually all of the current is now inactivated at −40 mV (see Figure 5C inset).

The P17T mutation shifts the steady state inactivation curve for K_V_2.1 to the right, with a corresponding shift in V_50_ to −23.7 mV (95% CI = −27.1 to −19.7 mV, Figures 5D, F) so that now some channels are available for activation even at −10 mV (see Figure 5D inset). The steady state inactivation for the P17T mutated channels co-expressed with K_V_9.3 is also shifted to the right, with a corresponding shift in V_50_ to −31.6 mV (95% CI = −37.7 to −24.4 mV, Figures 5E, F) so that again, some channels are available for activation even at −10 mV (see Figure 5E inset). This represents even more of a shift to the right in V_50_, for the K_V_2.1/K_V_9.3 co-expressed channels (28.8 mV) compared to K_V_2.1 channels alone (8.8 mV).

These shifts in the steady state inactivation curves can explain the increased currents seen for the mutated channels, when steps are made from a voltage close to the resting membrane potential (Figures 2, 3), since more mutated channels are available for activation at this voltage. Indeed, even at the highest voltage prepulse tested (+10 mV) there is still significant current available for activation in the P17T mutant K_V_2.1 channels whether alone or co-expressed with K_V_9.3.

### P17T mutation alters the effectiveness of the K_V_2.1 channel blocking toxin, guanxitoxin-1E

**FIGURE 6:**
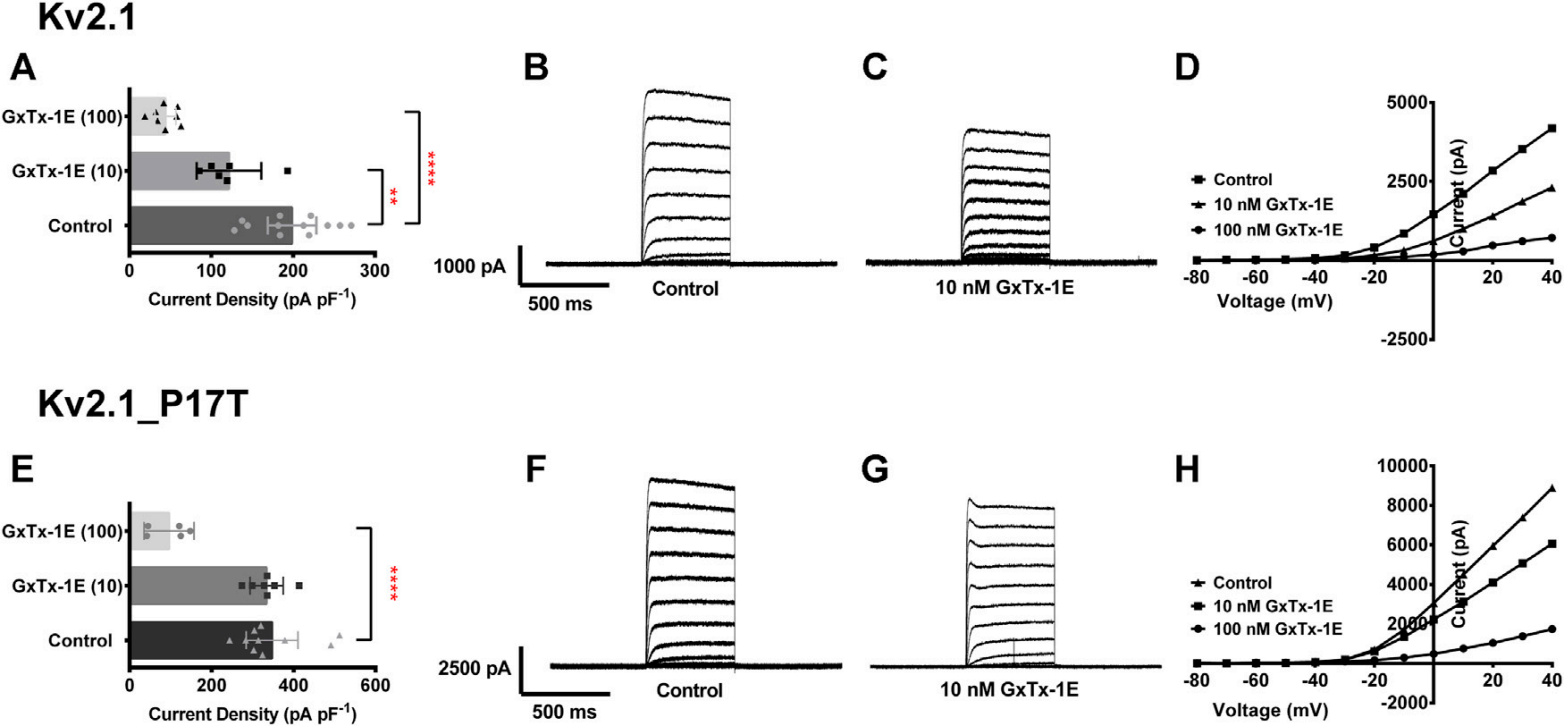
P17T mutation alters the effectiveness of the K_V_2.1 channel blocking toxin, guanxitoxin-1E (GxTx-1E). **(A)** Histogram of current density (pA pF-1) measured at +50 mV from individual cells, transiently expressing 500 ng of K_V_2.1-WT in control solution (*n* = 12), 10 nM GxTx-1E (*n* = 6) and 100 nM GxTx-1E (*n* = 8). Error bars represent 95% CI. Statistical significance was tested using a one-way ANOVA with a post-hoc Dunnett’s multiple comparisons test, *p* < .05 for ** and *p* < 0.001 for ****. **(B,C)** Average whole-cell current trace recorded using protocol A when expressing K_V_2.1-WT in the absence **(B)** and presence **(C)** of 10 nM GxTx-1E. **(D)** Representative current voltage relationships for cells expressing K_V_2.1-WT in control solution, 10 nM GxTx-1E and 100 nM GxTx-1E. **(E)** Histogram of current density (pA pF^−1^) measured at +50 mV from individual cells, transiently expressing 500 ng of K_V_2.1_P17T in control solution (*n* = 10), 10 nM GxTx-1E (*n* = 7) and 100 nM GxTx-1E (*n* = 5). Error bars represent 95% CI. Statistical significance was tested using a one-way ANOVA with a post-hoc Dunnett’s multiple comparisons test, *p* < 0.001 for ****. **(F,G)** Average whole-cell current trace recorded using protocol A when expressing K_V_2.1_P17T in the absence **(F)** and presence **(G)** of 10 nM GxTx-1E. **(H)** Representative current voltage relationships for cells expressing K_V_2.1_P17T in control solution, 10 nM GxTx-1E and 100 nM GxTx-1E.

The tarantula toxin, guanxitoxin-1E, is a known blocking agent of K_V_2.1 channels (Gupta et al., 2015), whose activity is influenced by the gating state of the K_V_2.1 channel (Navarro et al., 2019), being less effective at more depolarised voltages (Tilley et al., 2019). Given that the effect of this toxin is influenced by channel gating and that this is altered in the P17T mutated channels, we investigated whether the effectiveness of this toxin was also altered by the point mutation. Figure 6 shows that at a relatively low concentration (10 nM), the small but significant effect of the toxin on WT K_V_2.1 channels was not seen for the mutated channels. Guanxitoxin-1E (10 nM) reduced K_V_2.1-WT current from 199 ± 30 pA/pF (*n* = 12) to 122 ± 39 pA/pF (*n* = 6), (*p* < .05) but had no effect on K_V_2.1-P17T current (348 ± 63 pA/pF, *n* = 10, compared to 334 ± 40 pA/pF, *n* = 7 in the toxin). However, the toxin was effective on both WT and mutated K_V_2.1 channels at a 10-fold higher concentration (100 nM) showing that whilst the toxin has a reduced potency against the mutated channels, it can still block these channels at higher concentrations.

## Discussion

A patient has been identified with a novel mutation of KCNB1 (p.P17T; c49C>A) resulting in neurodevelopmental delay, speech apraxia, normal growth, normal EKG, and normal EEG. However, unlike 85% of patients identified with mutations in KCNB1, the patient has not developed seizures (Calhoun et al., 2017; de Kovel et al., 2017). As such, this mutation does not fit into the category of mutations known as Developmental and Epileptic Encephalopathies (DEE), whereby epileptic activity contributes to the observed developmental impairment (Scheffer et al., 2017). Other features of the phenotype, such as developmental delays and speech apraxia, however, are consistent with previous descriptions of patients with mutations of *KCNB1* (Bar et al., 2020).

Of the 55 distinct missense or loss of function mutations previously described (de Kovel et al., 2017; Bar et al., 2020; Xiong et al., 2022), 49 of them were located in the S1-S6 transmembrane segments of the protein, 5 were in the C terminus and just 1 of the mutations (a missense mutation, E43G) was located in the N terminus of the channel similar to P17T. Whilst the patient with this E43G mutation did suffer from epileptic seizures they also had a predicted damaging mutation in the GABA_A_ receptor *GABRA5* which may underlie many if not all of the clinical symptoms observed (Bar et al., 2020).

Characterization of the functional properties of the K_V_2.1 channel, showed that the P17T mutation enhances current through these homomeric channels without changing ion selectivity of the channel. The P17T mutation is even more effective at enhancing current through K_V_2.1/K_V_9.3 channel heteromeric channels. At least part of this effect is due to a change in the steady-state inactivation curves for these channels in the presence of the P17T mutation, so that more channels are available for activation at depolarised voltages, including the normal physiological resting potential.

In addition, the inactivation of P17T channels differs from that of WT (see Figure 2C, D), in that it exhibits two distinct phases of inactivation. As such, P17T channels may have a propensity to reside in a state from where channel opening is facilitated which would also contribute to the increased current seen. In addition, differences in state transitions between P17T and WT channels may contribute to the differential effects seen for guanxitoxin (see Figure 6 and below).

K_V_2.1 channels are highly expressed in the brain and are involved in regulating neuronal excitability. They have been demonstrated to act as homeostatic suppressors of heightened neuronal activity (Speca et al., 2014). The gain-of-function mutation described here, increases current, either when expressed as a homotetramer or when heteromerized with K_V_2.1-WT or silently inactive K_V_ channels, such as K_V_9.3. The GoF of these channels will act to put a brake on neuronal excitability by stabilising the resting membrane potential of the neurons where they are expressed. However, and paradoxically, if an excitatory stimulus is large enough to overcome this tonic reduced excitability, then the increased K_V_ current present will repolarise neuronal cells more quickly following an action potential, making them more readily available to fire repetitively should the stimulation be large enough.

This functional difference in channel properties, when contrasted with the known properties of other K_V_2.1 channel mutations, may explain why this patient does not suffer from epileptic seizures. Other *de novo* variants in K_V_2.1 act as dominant-negative influences to reduce WT currents. These include the mutations I199F which reduces channel availability (Calhoun et al., 2017), G401R which is a dominant negative mutation (Saitsu et al., 2015) and S347R, T374I, V378A, and G379R which all cause a loss of ion selectivity and reduced current density (Torkamani et al., 2014; Thiffault et al., 2015). These will result in a depolarised membrane potential and impaired membrane repolarisation leading to increased cellular excitability. In a high-throughput study of 17 variants (Kang et al., 2019), the majority of these (14/17) were shown to result in reduced channel function though a variety of molecular mechanisms, such as lower cell surface expression and/or alterations in the voltage dependence of channel activation and inactivation, all leading to a reduction in measured current. None of these mutations showed a gain of function phenotype (Kang et al., 2019) as described here. Similarly, deletion of K_V_2.1 channels in mice leads to neuronal and behavioural excitability (Speca et al., 2014).

It is perhaps surprising that although the novel GoF mutation described here does not lead to epileptic seizures it does lead to several symptoms such as developmental delay and speech apraxia. This may suggest that alteration in channel function in either direction away from the baseline activity seen in the absence of mutations can lead to the same clinical outcome, as seen for the K2P potassium channel TASK-3 in *KCNK9* imprinting syndrome (Cousin et al., 2022). A similar phenomenon has also been seen for the potassium channel BK (*KCNMA1*) where mutations of the channel lead to paroxysmal non-kinesigenic dyskinesia (PNKD3) with a broad spectrum of developmental and neurological phenotypes and seizures seen in a proportion of the patients (Liang et al., 2019). Some of these mutations are gain of function and others loss of function, albeit that gain of function mutations appear to have higher pathogenic potential (Park et al., 2022).

Alternatively, it may not the level of channel activity *per se* that leads to at least some of the common phenotypes observed across all mutations whether GOF or LOF, but rather, some other property of the mutated K_V_2.1 channels. In this respect it is of interest that K_V_2.1 channels have a non-conducting role in forming stable junctions between endoplasmic reticulum and plasma membranes and that this function produces effects that are independent of the channels roles in regulating membrane excitability (Maverick et al., 2021).

Interestingly, we have shown that the K_V_2.1 channel toxin, GxTx-1, is less effective at blocking P17T mutated channels compared to K_V_2.1-WT channels (Gupta et al., 2015), because the altered functional properties of the mutated channels interfere with the blocking action of the toxin (Navarro et al., 2019; Tilley et al., 2019). Other existing compounds which block K_V_2.1 channels may also have a different potency when used to block mutated channels, compared to WT channels, because of the altered functional properties of the channel. This has implications for any therapeutic approaches designed to reduce channel activity to resting levels, where it will be important that these approaches are tested against the mutated K_V_2.1 channels and not solely K_V_2.1-WT channels.

## Data Availability

The original contributions presented in the study are included in the article/Supplementary Materials, further inquiries can be directed to the corresponding author.

## References

[B1] Alexander S. P. H. Mathie A. Peters J. Veale E. L. Striessnig J. Kelly E. (2021). The concise guide to pharmacology 2021/2022: Ion channels. Br. J. Pharmacol. 178, S157–S245. 10.1038/sj.bjp.0706581 34529831

[B2] Bar C. Barcia G. Jennesson M. Le Guyader G. Schneider A. Mignot C. (2020). Expanding the genetic and phenotypic relevance of KCNB1 variants in developmental and epileptic encephalopathies: 27 new patients and overview of the literature. Hum. Mutat. 41, 69–80. 10.1002/humu.23915 31513310

[B3] Bocksteins E. (2016). Kv5, Kv6, Kv8, and Kv9 subunits: No simple silent bystanders. J. Gen. Physiol. 145, 105–125. 10.1085/jgp.201511507 PMC4727947 26755771

[B4] Calhoun J. D. Vanoye C. G. Kok F. George A. L. Kearney J. A. (2017). Characterization of a KCNB1 variant associated with autism, intellectual disability, and epilepsy. Neurol. Genet. 3, e198. 10.1212/NXG.0000000000000198 29264390 PMC5733249

[B5] Coonen L. Mayeur E. De Neuter N. Snyders D. J. Cuello L. G. Labro A. J. (2020). The selectivity filter is involved in the U-type inactivation process of Kv2.1 and Kv3.1 channels. Biophys. J. 118, 2612–2620. 10.1016/j.bpj.2020.03.032 32365329 PMC7231921

[B6] Cousin M. A. Veale E. L. Dsouza N. R. Tripathi S. Holden R. G. Arelin M. (2022). Gain and loss of TASK3 channel function and its regulation by novel variation cause KCNK9 imprinting syndrome. Genome Med. 14 (1), 62. 10.1186/s13073-022-01064-4 35698242 PMC9195326

[B7] Cunningham K. P. Holden R. G. Escribano-Subias P. M. Cogolludo A. Veale E. L. Mathie A. (2019). Characterization and regulation of wild-type and mutant TASK-1 two pore domain potassium channels indicated in pulmonary arterial hypertension. J. Physiol. 597 (4), 1087–1101. 10.1113/JP277275 30365877 PMC6376074

[B8] de Kovel C. G. F. Syrbe S. Brilstra E. H. Verbeek N. Kerr B. Dubbs H. (2017). Neurodevelopmental disorders caused by de novo variants in KCNB1 genotypes and phenotypes. JAMA Neurol. 74, 1228–1236. 10.1001/jamaneurol.2017.1714 28806457 PMC5710242

[B9] Gupta K. Zamanian M. Bae C. Milescu M. Krepkiy D. Tilley D. C. (2015). Tarantula toxins use common surfaces for interacting with Kv and ASIC ion channels. eLife 4, e06774. 10.7554/eLife.06774 25948544 PMC4423116

[B10] Johnson B. Leek A. N. Tamkun M. M. (2019). Kv2 channels create endoplasmic reticulum/plasma membrane junctions: A brief history of Kv2 channel subcellular localization. Channels 13, 88–101. 10.1080/19336950.2019.1568824 30712450 PMC6380216

[B11] Ju M. Stevens L. Leadbitter E. Wray D. (2003). The roles of N- and C- terminal determinants in the activation of the Kv2.1 potassium channel. J. Biol. Chem. 278, 12769–12778. 10.1074/jbc.M212973200 12560340

[B12] Kang S. K. Vanoye C. G. Misra S. N. Echevarria D. M. Calhoun J. D. O'Connor J. B. (2019). Spectrum of Kv2.1 dysfunction in KCNB1-associated neurodevelopmental disorders. Ann. Neurol. 86, 899–912. 10.1002/ana.25607 31600826 PMC7025436

[B13] Kerschensteiner D. Monje F. Stocker M. (2003). Structural determinants of the regulation of the voltage-gated potassium channel Kv2.1 by the modulatory α-subunit Kv9.3. J. Biol. Chem. 278, 18154–18161. 10.1074/jbc.M213117200 12642579

[B14] Kerschensteiner D. Stocker M. (1999). Heteromeric assembly of Kv2.1 with Kv9.3: Effect on the state dependence of inactivation. Biophys. J. 77, 248–257. 10.1016/S0006-3495(99)76886-4 10388754 PMC1300326

[B15] Klemic K. G. Kirsch G. E. Jones S. W. (2001). U-type inactivation of Kv3.1 and *shaker* potassium channels. Biophys. J. 81, 814–826. 10.1016/S0006-3495(01)75743-8 11463627 PMC1301555

[B16] Klemic K. G. Shieh C-C. Kirsch G. E. Jones S. W. (1998). Inactivation of Kv2.1 potassium channels. Biophys. J. 74, 1779–1789. 10.1016/S0006-3495(98)77888-9 9545040 PMC1299522

[B17] Kramer J. W. Post M. A. Brown A. M. Kirsch G. E. (1998). Modulation of potassium channel gating by coexpression of Kv2.1 with regulatory Kv5.1 or Kv6.1 α-subunits. Am. J. Physiol. 274, C1501–C1510. 10.1152/ajpcell.1998.274.6.C1501 9696692

[B18] Kurata H. T. Doerksen K. W. Eldstrom J. R. Rezazadeh S. Fedida D. (2005). Separation of P/C- and U-type inactivation pathways in Kv1.5 potassium channels. J. Physiol. 568, 31–46. 10.1113/jphysiol.2005.087148 16020465 PMC1474772

[B19] Kurata H. T. Fedida D. (2006). A structural interpretation of voltage-gated potassium channel inactivation. Prog. Biophys. Mol. Biol. 92, 185–208. 10.1016/j.pbiomolbio.2005.10.001 16316679

[B20] Kurata H. T. Soon G. S. Eldstrom J. R. Lu G. W. K. Steele D. F. Fedida D. (2002). Amino-terminal determinants of U-type inactivation of voltage-gated K^+^ channels. J. Biol. Chem. 277, 29045–29053. 10.1074/jbc.M111470200 12021261

[B21] Liang L. Li X. Moutton S. Schrier Vergano S. A. Cogne B. Saint-Martin A. (2019). De novo loss-of-function KCNMA1 variants are associated with a new multiple malformation syndrome and a broad spectrum of developmental and neurological phenotypes. Hum. Mol. Gen. 28, 2937–2951. 10.1093/hmg/ddz117 31152168 PMC6735855

[B22] Lvov A. Chikvashvili D. Michaelevski I. Lota I. (2008). VAMP2 interacts directly with the N terminus of Kv2.1 to enhance channel inactivation. Pflugers Arch. 456, 1121–1136. 10.1007/s00424-008-0468-7 18542995

[B23] Mathie A. Veale E. L. Holden R. G. (2021). Heterologous expression of ion channels in mammalian cell lines. Methods Mol. Biol. 2188, 51–65. 10.1007/978-1-0716-0818-0_3 33119846

[B24] Maverick E. E. Leek A. N. Tamkun M. M. (2021). Kv2-channel-AMIGOβ-subunit assembly modulates both channel function and cell adhesion molecule surface trafficking. J. Cell Sci. 134 (12), jcs256339. 10.1242/jcs.256339 34137443 PMC8255027

[B25] Misonou H. Mohapatra D. P. Trimmer J. S. (2005). Kv2.1: A voltage-gated K+ channel critical to dynamic control of neuronal excitability. Neurotoxicol 26, 743–752. 10.1016/j.neuro.2005.02.003 15950285

[B26] Navarro M. A. Milescu L. S. Milescu M. (2019). Unlocking the gating mechanism of Kv2.1 using guangxitoxin. J. Gem. Physiol. 151, 275–278. 10.1085/jgp.201812254 PMC6400516 30563879

[B27] O’Dwyer S. C. Palacio S. Matsumoto C. Guarina L. Klug N. R. Tajada S. (2020). Kv2.1 channels play opposing roles in regulating membrane potential, Ca2+ channel function, and myogenic tone in arterial smooth muscle. PNAS U. S. A. 117, 3858–3866. 10.1073/pnas.1917879117 PMC7035623 32015129

[B28] Park S. M. Roache C. E. Iffland P. H. Moldenhauer H. J. Matychak K. K. Plante A. E. (2022). BK channel properties correlate with neurobehavioral severity in three KCNMA1-linkedchannelopathy mouse models. Elife 11, e77953. 10.7554/eLife.77953 35819138 PMC9275823

[B29] Patel A. J. Lazdunski M. Honoré E. (1997). Kv2.1/Kv9.3, a novel ATP-dependent delayed-rectifier K+ channel in oxygen-sensitive pulmonary artery myocytes. EMBO J. 16, 6615–6625. 10.1093/emboj/16.22.6615 9362476 PMC1170266

[B30] Post M. A. Kirsch G. E. Brown A. M. (1996). Kv2.1 and electrically silent Kv6.1 potassium channel subunits combine and express a novel current. FEBS Lett. 399, 177–182. 10.1016/s0014-5793(96)01316-6 8980147

[B31] Retterer K. Juusola J. Megan T. Vitazka P. Millan F. Gibellini F. (2016). Clinical application of whole-exome sequencing across clinical indications. Genet. Med. 18, 696–704. 10.1038/gim.2015.148 26633542

[B32] Saitsu H. Akita T. Tohyama J. Goldberg-Stern H. Kobayashi Y. Cohen R. (2015). De novo KCNB1 mutations in infantile epilepsy inhibit repetitive neuronal firing. Sci. Rep. 5, 15199. 10.1038/srep15199 26477325 PMC4609934

[B33] Salinas M. Duprat F. Heurteaux C. Hugnot J. P. Lazdunski M. (1997). New modulatory alpha subunits for mammalian Shab K+ channels. J. Biol. Chem. 272, 24371–24379. 10.1074/jbc.272.39.24371 9305895

[B34] Scheffer I. E. Berkovic S. Capovilla G. Connolly M. B. French J. Guilhoto L. (2017). ILAE classification of the epilepsies: Position paper of the ILAE commission for classification and terminology. Epilepsia 58, 512–521. 10.1111/epi.13709 28276062 PMC5386840

[B35] Shah N. H. Aizenman E. (2014). Voltage-gated potassium channels at the crossroads of neuronal function, ischemic tolerance, and neurodegeneration. Transl. Stroke Res. 5, 38–58. 10.1007/s12975-013-0297-7 24323720 PMC3946373

[B36] Speca D. J. Ogata G. Mandikian D. Bishop H. I. Wiler S. W. Eum K. (2014). Deletion of the Kv2.1 delayed rectifier potassium channel leads to neuronal and behavioral hyperexcitability. Genes Brain Behav. 13, 394–408. 10.1111/gbb.12120 24494598 PMC4077602

[B37] Stocker M. Hellwig M. Kerschensteiner D. (1999). Subunit assembly and domain analysis of electrically silent K+ channel alpha-subunits of the rat Kv9 subfamily. J. Neurochem. 72, 1725–1734. 10.1046/j.1471-4159.1999.721725.x 10098883

[B38] Thiffault I. Speca D. J. Austin D. C. Cobb M. M. Eum K. S. Safina N. P. (2015). A novel epileptic encephalopathy mutation in KCNB1 disrupts Kv2.1 ion selectivity, expression, and localization. J. Gen. Physiol. 146, 399–410. 10.1085/jgp.201511444 26503721 PMC4621747

[B39] Tilley D. C. Angueyra J. M. Eum K. S. Kim H. Chao L. H. Peng A. W. (2019). The tarantula toxin GxTx detains K^+^ channel gating charges in their resting conformation. J. Gen. Physiol. 151, 292–315. 10.1085/jgp.201812213 30397012 PMC6400525

[B40] Torkamani A. Bersell K. Jorge B. S. Bjork R. L. Friedman J. R. Bloss C. S. (2014). De novo KCNB1 mutations in epileptic encephalopathy. Ann. Neurol. 76, 529–540. 10.1002/ana.24263 25164438 PMC4192091

[B41] Veale E. L. Al Moubarak E. Bajaria N. Omoto K. Cao L. Tucker S. J. (2014b). Influence of the N-terminus on the biophysical properties and pharmacology of TREK-1 potassium channels. Mol. Pharmacol. 85, 671–681. 10.1124/mol.113.091199 24509840

[B42] Veale E. L. Hassan M. Walsh Y. Al-Moubarak E. Mathie A. (2014a). Recovery of current through mutated TASK3 potassium channels underlying birk barel syndrome. Mol. Pharmacol. 85, 397–407. 10.1124/mol.113.090530 24342771

[B43] Xiong J. Liu Z. Chen S. Kessi M. Chen B. Duan H. (2022). Correlation analyses of clinical manifestations and variant effects in *KCNB1*-related neurodevelopmental disorder. Front. Pediatr. 9, 755344. 10.3389/fped.2021.755344 35071126 PMC8767024

[B44] Yellen G. (2002). The voltage-gated potassium channels and their relatives. Nature 419, 35–42. 10.1038/nature00978 12214225

