# Supplementary material for: A KCNB1 gain of function variant causes developmental delay and speech apraxia but not seizures
Source: Front Pharmacol. 2022 Dec 21;13:1093313. doi: 10.3389/fphar.2022.1093313 (PMC9810754; doi:10.3389/fphar.2022.1093313)
Supplement: Supplementary file 1 [file DataSheet1.PDF]

Supplementary Material

A KCNB1 gain of function variant causes developmental delay and speech apraxia but not seizures.

Emma L Veale, Alessia Golluscio, Katheryn Grand, John M Graham Jr, Alistair Mathie

*** Correspondence:** Alistair Mathie: a.a.mathie@kent.ac.uk

Supplementary Figure 1.

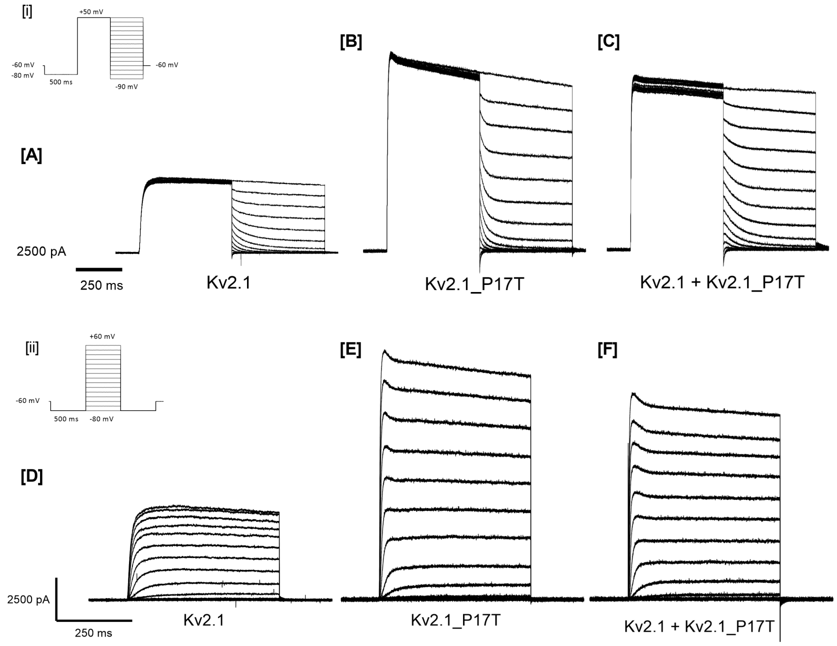

K_V2.1_P17T channel subunits are dominant positive when co-expressed with K_V2.1 channel subunits.

[A] Average whole-cell current trace recorded using protocol B (inset [i]) when expressing Kv2.1-WT. [B] Average whole-cell current trace recorded using protocol B (inset [i]) when expressing Kv2.1-P17T [C] Average whole-cell current trace recorded using protocol B (inset [i]) when co-

expressing Kv2.1-WT and Kv2.1-P17T (1:1). [D] Average whole-cell current trace recorded using protocol A (inset [ii]) when expressing Kv2.1-WT. [E] Average whole-cell current trace recorded using protocol A (inset [ii]) when expressing Kv2.1-P17T. [F] Average whole-cell current trace recorded using protocol A (inset [ii]) when co-expressing Kv2.1-WT and Kv2.1-P17T.
